# Purification, Characterization, cDNA Cloning, and Bioinformatic Analysis of Zinc-Binding Protein from *Magallana hongkongensis*

**DOI:** 10.3390/molecules29040900

**Published:** 2024-02-18

**Authors:** Citing Chen, Wan Li, Jialong Gao, Wenhong Cao, Xiaoming Qin, Huina Zheng, Haisheng Lin, Zhongqin Chen

**Affiliations:** 1 College of Food Science and Technology, Guangdong Ocean University, Zhanjiang 524088, China; 13414955284@163.com (C.C.); m18944942363@163.com (W.L.); cwenhong@gdou.edu.cn (W.C.); qxmspkx2016@163.com (X.Q.); zhenghn@gdou.edu.cn (H.Z.); haishenglin@163.com (H.L.); chenzhongqin@gdou.edu.cn (Z.C.); 2 Guangdong Provincial Key Laboratory of Aquatic Products Processing and Safety, Guangdong Province Engineering Laboratory for Marine Biological Products, Key Laboratory of Advanced Processing of Aquatic Product of Guangdong Higher Education Institution, Zhanjiang 524088, China

**Keywords:** *Magallana hongkongensis*, zinc-binding protein, cDNA cloning, carbonic anhydrase

## Abstract

Oysters contain significant amounts of the zinc element, which may also be found in their proteins. In this study, a novel zinc-binding protein was purified from the mantle of the oyster *Magallana hongkongensis* using two kinds of gel filtration chromatograms. Sodium dodecyl sulfate–polyacrylamide gel electrophoresis (SDS-PAGE) showed that its molecular weight was approximately 36 kDa. The protein identified by the Q-Exactive mass spectrometer shared the highest sequence identity with carbonic anhydrase derived from *Crassostrea gigas* concerning amino acid sequence similarity. Based on homologous cloning and RACE PCR, the full-length cDNA of carbonic anhydrase from *Magallana hongkongensis* (designated as MhCA) was cloned and sequenced. The cDNA of MhCA encodes a 315-amino-acid protein with 89.74% homology to carbonic anhydrase derived from *Crassostrea gigas*. Molecular docking revealed that the two zinc ions primarily form coordination bonds with histidine residues in the MhCA protein. These results strongly suggest that MhCA is a novel zinc-binding protein in *Magallana hongkongensis*.

## 1. Introduction

Zinc is an essential micronutrient involved in biological processes as a structural, catalytic, and intracellular signal element [1,2]. Zinc deficiency has become a global health problem, with approximately 17% of the world’s population at risk for zinc deficiency [3], which can result in a wide range of physiological defects, such as growth retardation, skin lesions, and immune disorders, and can be fatal in severe cases [4,5]. Zinc deficiency may be inherited from mutations in zinc transporters, or it may be caused by inadequate zinc intake and absorption [6,7]. Inadequate zinc absorption is a major cause of zinc deficiency and can be improved by dietary fortification or supplementation.

For years, researchers have focused on developing techniques and products, such as zinc salt supplementation and dietary fortification, to increase zinc intake and combat zinc deficiency. However, multiple concerns have been raised regarding the efficacy and safety of the use of inorganic and organic zinc salts, which can cause intestinal absorption instability, irritation, and disease [8]. Compared with zinc salts, hydrolysates and peptides of zinc-chelating proteins have been produced from different dietary proteins, which have been shown to have superior stability and bioavailability [9,10].

Dietary zinc can be obtained from a wide range of foods, including meat, seafood, mushrooms, and plants. The highest zinc concentrations were found in oysters compared to foods of animal origin, fish, and shellfish [11]. The zinc concentrations in oysters can be as high as 1–5‰ of tissue dry weight, which is 10 to 50 times higher than that of human beings, abalone, and scallops [12,13].

In organisms, cellular zinc is available in four pools. Zinc binding predominantly occurs with metallothioneins (MTs), which make up 5–15% of the overall cellular zinc pool and exhibit the lowest affinity towards zinc. Second, it may be compartmentalized into intracellular vesicles for zinc storage and as a supply for zinc-dependent proteins, which are mediated by zinc transporters. Third, it can bind tightly to metalloproteins as a structural component, or to metalloenzymes as a cofactor. Fourth, it was maintained at a very low concentration in the form of cytosolic-free zinc [14]. The current number of publicly released MT gene sequences (both genomic and mRNA sequences; https://www.ncbi.nlm.nih.gov/genbank/, accessed on 5 February 2023) of the mollusk species have outgrown over 100 by excluding the partial sequences or untrimmed ESTs. And 28 zinc transporter genes have been identified in pacific oyster *C. gigas* [13], which are transmembrane proteins that regulate zinc content via mobilizing zinc across cellular and intracellular membranes [15]. Free zinc is unstable and requires a zinc-binding protein to maintain zinc efficiency [16]. In metalloproteins or metalloenzymes, zinc plays a crucial role in their structure [17]. To date, more than 3000 proteins have been identified that have Zn^2+^ as a cofactor to play an important role in life, one of which is carbonic anhydrase [18,19].

*Magallana hongkongensis*, the Hong Kong oyster, is a commercially valuable aquaculture species and is mainly located on the northern coast of the South China Sea. Oysters have long been considered as an excellent food for the supplementation of zinc, due to their high zinc content. Zinc mainly exists in oysters in the form of free zinc, which is unstable, and zinc-binding protein, which can maintain zinc efficiency. However, the protein associated with zinc is unclear in oysters. Therefore, in the current study, a novel zinc-binding protein from the mantle of *M. hongkongensis* was isolated and purified using gel filtration. The protein was identified using a Q-Exactive mass spectrometer and MASCOT search. The full-length cDNA sequence of the target protein was obtained based on homologous cloning and the RACE PCR method. Finally, the binding mode between the protein and zinc ions was analyzed using molecular docking.

## 2. Results

### 2.1. The Distribution of Zinc Content in Tissues

**Figure 1:**
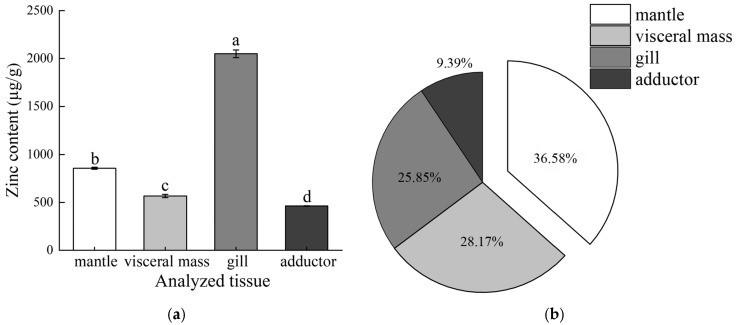
(**a**) Zinc contents in analyzed tissues of *M. hongkongensis*. (**b**) The proportions of zinc content in analyzed tissues accounted for them in all the soft tissues of *M. hongkongensis*. Data are presented as mean ± S.D. (*n* = 3). Bars bearing different letters are significantly different (*p* < 0.05; Tukey’s test).

The zinc contents and proportions of oyster tissues (including mantle, visceral mass, gill, and adductor) were measured using ICP-MS (Figure 1). Based on the weight dried of different tissues, the zinc content was 856.12 ± 10.49 µg/g, 567.42 ± 17.49 µg/g, 2049.52 ± 40.22 µg/g, and 462.27 ± 2.65 µg/g in mantle, visceral mass, gill, and adductor, respectively (Figure 1a). Tissues were ranked in descending order of zinc content levels as follows: gill > mantle > visceral mass > adductor (*p* < 0.05). Based on the dry weight of different tissues, the proportion of zinc content was 36.58 ± 0.33%, 28.17 ± 0.87%, 25.85 ± 0.6%, and 9.38 ± 0.11% in the mantle, visceral mass, gill, and adductor, respectively (Figure 1b). Tissues were ranked in descending order of zinc content proportion levels as follows: mantle > visceral mass > gill > adductor (*p* < 0.05).

### 2.2. Isolation and Purification of Zinc-Binding Protein

**Figure 2:**
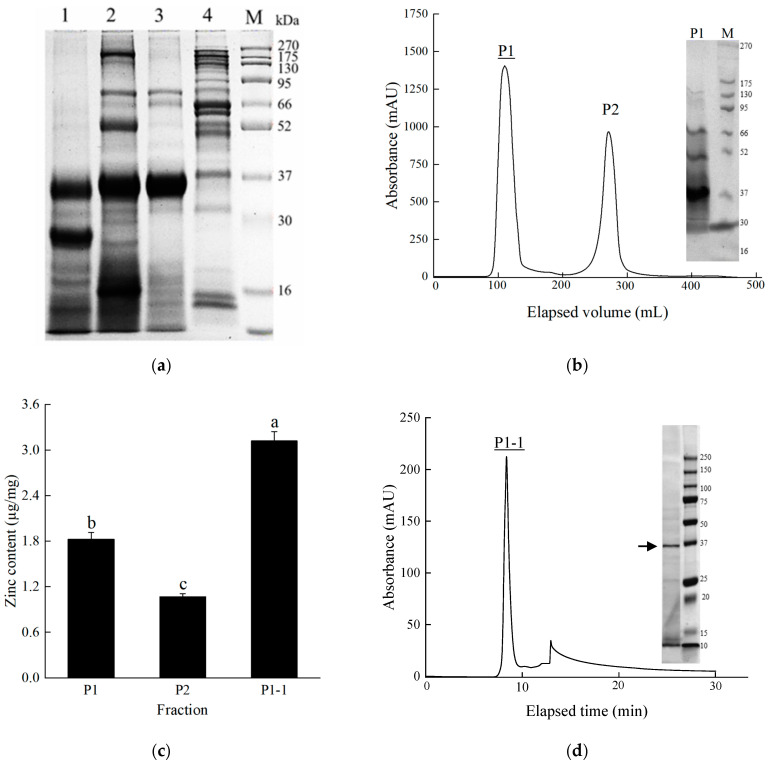
(**a**) Protein analysis (aliquot 10 µg) by 10% SDS-PAGE (M: marker, Lane 1 to 4: WSPs in visceral mass, mantle, gill, and adductor). (**b**) Gel filtration chromatogram of fractions eluted from a Superdex 200 column detected by UV detector at 280 nm, and 10% SDS-PAGE analysis of fraction P1 collected from gel filtration chromatography. (**c**) Zinc contents of fractions P1, P2, and P1-1. The fractions P1 and P2 were collected from Superdex 200 gel filtration chromatography, and fraction P1-1 was collected from TSKgel G2000SWXL gel filtration chromatography. Bars bearing different letters are significantly different (*p* < 0.05; Tukey’s test). (**d**) Gel filtration chromatogram of fractions eluted from a TSKgel G2000SWXL column detected by UV detector at 280 nm, and 10% SDS-PAGE analysis of fraction P1-1 collected from gel filtration chromatography. Arrow denotes target protein.

Figure 2a shows the result of electrophoretic analysis of water-soluble proteins (WSPs) obtained from visceral mass, mantle, gill, and adductor in the oyster. Sodium dodecyl sulfate–polyacrylamide gel electrophoresis (SDS-PAGE) analysis revealed that molecular weight (MW) distribution profiles were expressed differently in different tissues. The WSP in the visceral mass exhibited low MWs, predominantly ranging from 37 to 10 kDa. MWs from the WSP were high and widely distributed in the mantle, mostly in the range of 270 kDa, 80–52 kDa, and 37–10 kDa. A few bands with MWs of 80–66 and 37 kDa were observed in the WSP from the gill. The MWs showed a broader distribution of WSP in the adductor (270–10 kDa). One of the most striking similarities was the appearance of a band at approximately 36 kDa that was dark and wide in all WSPs originating from the visceral mass, mantle, and gill. In contrast, there was no obvious bright band at 36 kDa found in adductor WSP. Considering the high proportion of zinc in the mantle and the low proportion in the adductor (Figure 1b), crude WSP from the mantle was chosen for further purification.

The Superdex 200 gel-filtration chromatography system was used to purify the crude WSP from the mantle, and the eluted fractions were tracked by their absorbance at 280 nm. Two main peaks (P1 and P2) were eluted as indicated in Figure 2b. The contents of zinc in these two fractions were determined to be 1.82 ± 0.09 µg/mg and 1.06 ± 0.04 µg/mg, respectively. The P1 fraction was considered to contain the majority of zinc-binding proteins in this analysis. The SDS-PAGE analysis revealed that P1 consisted of four or more bands with MWs of approximately 66, 50, 36, and 30 kDa (Figure 2b). The pooled P1 fraction underwent additional purification performed using a TSKgel G2000SWXL high-performance liquid chromatography system for the purpose of purifying zinc-binding proteins. At 280 nm, the profiles displayed a major and minor peak. The major peak (P1-1) exhibited the utmost concentration of zinc (3.12 ± 0.12 μg/mg) (Figure 2c) and the clear band with MW of approximately 36 kDa (Figure 2d).

### 2.3. Identification of Zinc-Binding Protein

The identification of the 36-kDa protein was accomplished through the utilization of a Q-Exactive mass spectrometer. The mass figures varied between 1024.4825 and 1630.8355. Sequencing was performed on fragments 1 to 4 of these digested peptides (Table 1). The zinc-binding protein of *M. hongkongensis* was paired with a protein called carbonic anhydrase (CA) (gene bank accession number: XP-011434938), achieving a top score of 92.

**Table 1:** Amino acid sequences of 36-kDa protein peptide fragments identified by Q-Exactive Mass Spectrometer.

Observed Peptide (*m*/*z*)	Calculated Peptide Mass	Sequence	Corresponding Position of MhCA ^1^
513.2501	1024.4825	KYGDLSNAASKE	143–154
643.8151	1285.6105	RTAQF**H**F**H**WGRS	106–117
795.4011	1588.78	RFRTAQF**H**F**H**WGRS	104–117
816.4278	1630.8355	RYPLEL**H**IVNYNEKY	130–144

^1^ The number of amino acid residues of MhCA refers to Figure 3. Zinc-binding sites are shown in bold.

**Figure 3:**
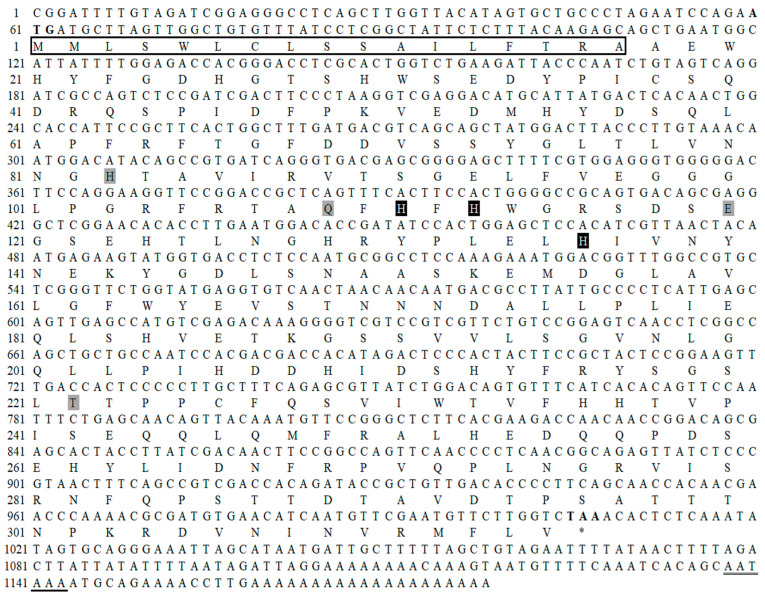
The nucleotide and deduced amino acid sequence of MhCA. Start codon (ATG) and stop codon (TAA) are shown in bold. Position, not encoding amino acids, is depicted as an asterisk (*). Polyadenylation signals (AATAAA) are underlined. Three zinc-binding sites and active sites (His^111^, His^113^, and His^136^) are shown in white characters on black background. Four active sites (His^83^, Gln^109^, Glu^120^, and Thr^222^) are shown on a gray background. The signal peptide (^1^MMLSWLCLSSAILFTRA^17^) is boxed.

### 2.4. cDNA Cloning and Sequence Characterization of MhCA

A partial cDNA sequence of 274 bp was amplified using primers (CA-F1 and CA-R1) derived from a conserved region in the CA sequences of mollusk. Using Blast analysis, this 274 bp region was found to have 92% similarity to *C. gigas* CA [20]. To obtain the full cDNA sequence, 5′- and 3′-end amplification were performed using primers (CA-F2, CA-F3, CA-R2, and CA-R3) designed from the partial sequence obtained. The product lengths were 618 bp (5′-end amplification) and 811 bp (3′-end amplification), respectively. After the overlap of the three fragments, a full-length cDNA sequence (1178 bp) of *M. hongkongensis* encoding the CA gene was obtained, which was confirmed using two primers (CA-F4 and CA-R4). The full-length cDNA of MhCA was 1178 bp, containing a 5′ untranslated region of 59 bp, a 3′ untranslated region of 171 bp, and an open reading frame (ORF) of 948 bp. The calculated molecular mass of the mature protein (315 amino acids) was 35.67 kDa with an estimated isoelectric point of 5.38 (gene bank accession no. OQ398502). Based on the search for conserved domains, the signature of alpha-carbonic anhydrases was found in MhCA, suggesting that the gene MhCA is a member of the carbonic anhydrase family. The full-length cDNA sequence revealed 90.89% similarity to CA2 from *C. gigas*, with the highest gene similarity. In the N-terminal of MhCA, there was a signal peptide with 17 amino acid residues (^1^MMLSWLCLSSAILFTRA^17^). It contained seven active amino acid residues (His^83^, Gln^109^, His^111^, His^113^, Glu^120^, His^136^, and Thr^222^) in the CA domain (Figure 3), including three zinc ion-binding sites consisting of three histidine residues (His^111^, His^113^, and His^136^).

**Figure 4:**
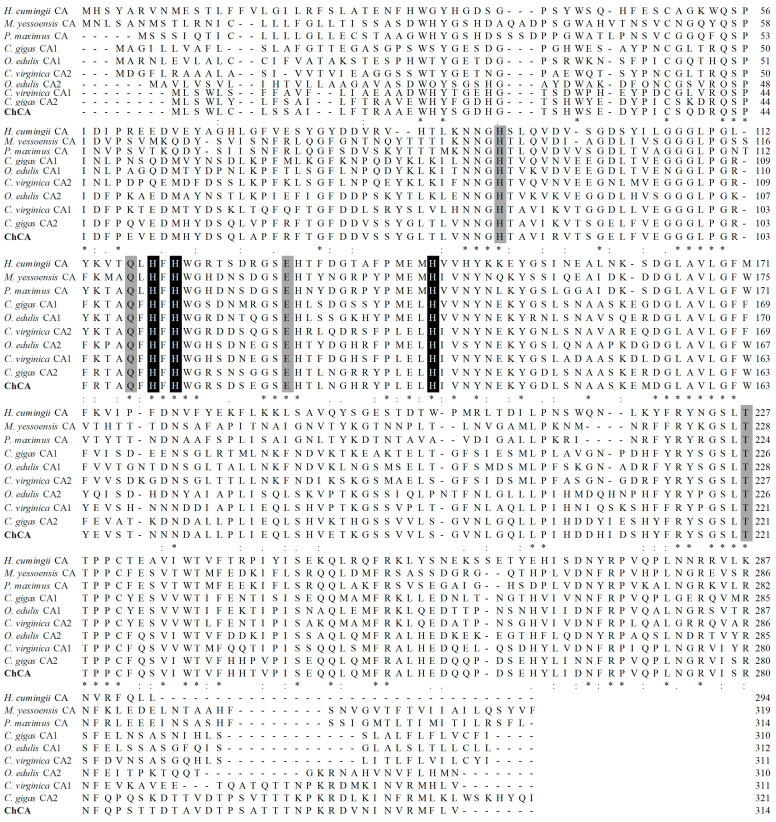
Clustal W alignment of CA sequences from *M. hongkongensis* against nine selected CA sequences. Sequence similarity is delineated as identical (*), strongly similar (:), and weakly similar (.). Three zinc-binding sites and active sites (His^111^, His^113^, and His^136^) are shown in white characters on black background. Four active sites (His^83^, Gln^109^, Glu^120^, and Thr^222^) are shown on a gray background. MhCA is shown in bold.

Similarity analysis indicated that the deduced amino acid sequence of MhCA shares a high degree of conservation with other reported CAs (Figure 4). MhCA presented 89.74% identity with *C. gigas* CA2 (XP-011434938), 70.06% with *C. virginica* CA1 (XP-022312365), 52.08% with *C. gigas* CA1 (XP-011455036), 50.35% with *Ostrea edulis* CA1 (XP-048770910), 47.45% with *Pecten maximus* CA (XP-033747437), 46.32% with *Mizuhopecten yessoensis* CA (XP-021373119), and 42.86% with *Hyriopsis cumingii* CA (AYN64225). Based on multiple alignments, three histidine amino acid residues (His^111^, His^113^, and His^136^), as well as seven active sites (His^83^, Gln^109^, His^111^, His^113^, Glu^120^, His^136^, and Thr^222^) were highly conserved across all the CAs analyzed.

**Figure 5:**
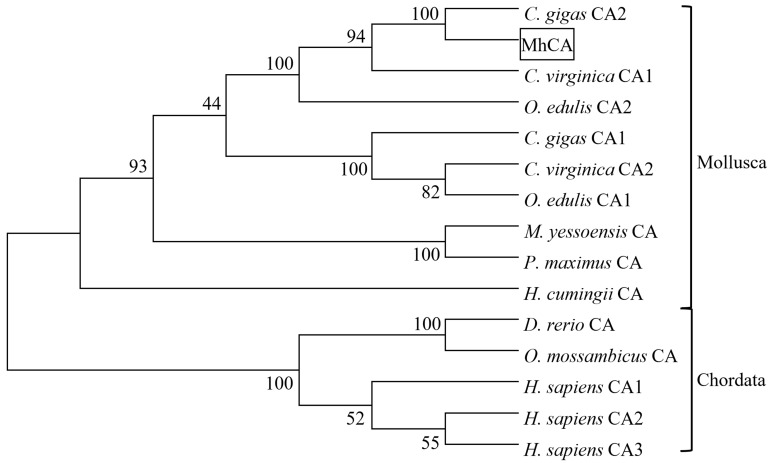
Consensus neighbor-joining tree of protein sequence from *M. hongkongensis* and other selected CA sequences. Aligned sequences were bootstrapped 1000 times and the numbers at the forks indicate the bootstrap proportions. MhCA is shown in the box. The amino acid sequences were derived from the GenBank under the following accession numbers (in parentheses): *C. gigas* CA2 (XP-011434938), *C. virginica* CA1 (XP-022312365), *O. edulis* CA2 (XP-048778796), *O. edulis* CA1 (XP-048770910), *C. gigas* CA1 (XP-011455036), *C. virginica* CA2 (XP-022337157), *M. yessoensis* CA (XP-021373119), *Pecten maximus* CA (XP-033747437), *H. cumingii* CA (AYN64225), *Danio rerio* CA (NP_571185), *Homo sapiens* CA1 (NP_001122301), *H. sapiens* CA2 (KAI2550532), *H. sapiens* CA3 (NP_005172), *Oreochromis mossambicus* CA (AAQ89896).

A phylogenetic tree constructed on the basis of the whole amino acid sequence of the CA gene revealed that there were two distinct clusters in this tree. Within the first cluster were mollusk CA sequences. The second cluster was chordate CA sequences (Figure 5). And the deduced amino acid sequence of MhCA has the closest homology to the amino acid sequence of CA2 from *C. gigas*.

**Figure 6:**
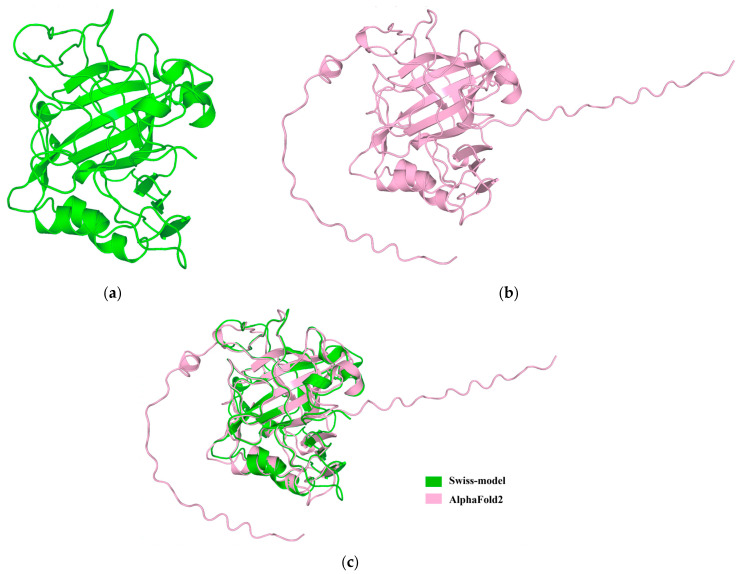
(**a**) The 3D structure of MhCA predicted by Swiss-model, utilizing the human CA II (4PXX) as a template. (**b**) The 3D structure of MhCA predicted by AlphaFold2. (**c**) The overlapping of two structures in PyMOL software.

Utilizing the Swiss-model, the predicted 3D structure of the MhCA protein was established (Figure 6a). Chosen as a template, the human CA II (PDB code: 4PXX) stands out for its top Global Model Quality Estimate value (0.67) in Swiss-model (http://swissmodel.expasy.org, accessed on 5 February 2023) and its high affinity, attributed to its unique state of containing highly thermal stabilized variant and non-sulfur-containing inhibitor [21]. The adequacy of the created model was evaluated based on the standard stereochemical settings of Procheck and Verfiy3D. The predicted structure of the MhCA protein revealed that 98.7% of its residues were in the allowed region, while the Ramachandran plot indicated an absence of amino acid residues in the disallowed region. Furthermore, the predicted structure of protein MhCA showed that 91.76% of its amino acid residues achieved or surpassed a score of 0.2, and over 80% of these residues satisfied the criteria of the assessment process. By employing AlphaFold2, the 3D structure of the MhCA protein was successfully predicted, as shown in Figure 6b. To validate the two predicted structures, the root mean square deviation (RMSD) value was utilized to assess the extent of their variance. A smaller value indicates greater similarity. In the PyMOL 2.2.0 software, these two formations were superimposed (Figure 6c). Post-calculation, the RMSD measurement stood at 0.18 nm, signifying a high similarity between the two structures.

**Figure 7:**
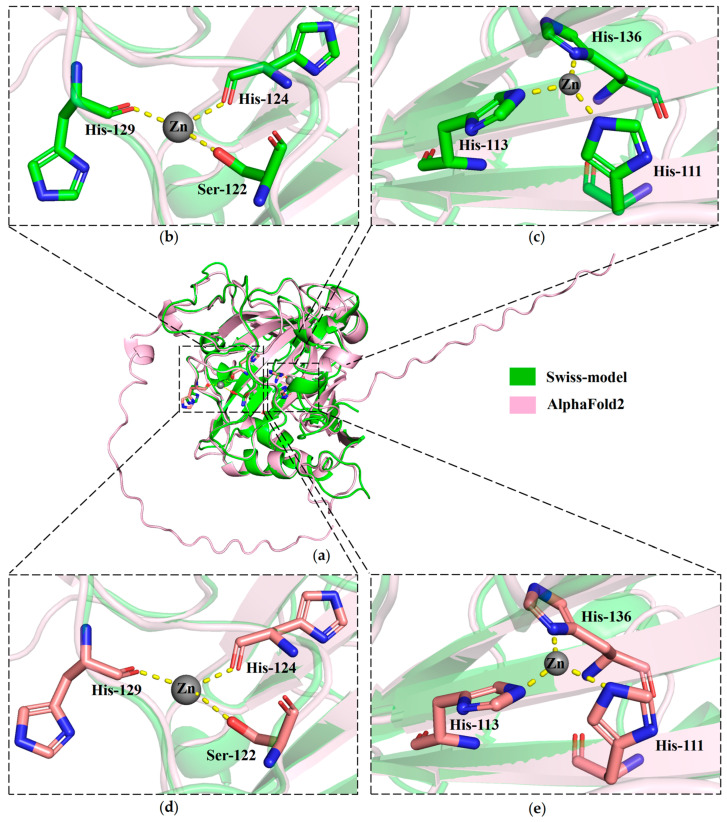
Molecular docking analysis of MhCA with zinc ions. (**a**) Molecular docking analysis of MhCA’s 3D structures predicted by Swiss-model and AlphaFold2. (**b**) Close-up view of zinc ion binding sites between the Swiss-model-predicted MhCA’s structure and one zinc ion. (**c**) Close-up view of zinc ion binding sites between the Swiss-model-predicted MhCA’s structure and the other zinc ion. (**d**) Close-up view of zinc ion-binding sites between the AlphaFold2 predicted MhCA’s structure and one zinc ion. (**e**) Close-up view of zinc ion-binding sites between the AlphaFold2 predicted MhCA’s structure and the other zinc ion. The zinc ion is represented by the gray sphere. Zn-coordinating amino acid residues (His^111^, His^113^, Ser^122^, His^124^, His^129^, and His^136^) are shown as sticks (stick model). The yellow dotted lines express the coordination between the zinc ion and the amino acid residues.

The molecular docking method was employed to analyze the binding sites, utilizing two protein structures as receptors and zinc ions as ligands. Findings indicated that each protein structure exhibited dual modes of interaction with ligands, with their binding energy being below zero (Figure 7a). This indicates a strong binding affinity between two zinc ions and the protein MhCA. Employing the Swiss-model-predicted MhCA’s 3D structure with zinc ions as receptors and ligands, it was found that one zinc ion attached to a serine residue and two histidine residues (Ser^122^, His^124^, and His^129^) through coordination bonds (Figure 7b), while the other zinc ion was linked to three histidine residues (His^111^, His^113^, and His^136^) via coordination bonds (Figure 7c). Using the AlphaFold2-predicted MhCA’s 3D structure as the receptor and zinc ions as the ligand, it was found that one zinc ion attached to serine and two histidine residues (Ser^122^, His^124^, and His^129^) via coordination bonds (Figure 7d), while the other zinc ion was connected to three histidine residues (His^111^, His^113^, and His^136^) via coordination bonds (Figure 7e). The outcomes stayed uniform.

## 3. Discussion

The zinc content in the oyster mantle was lower than in gill, but the proportion of zinc content in the mantle was higher than that in the other tissues. In the present study, a high level of zinc was determined in the oyster, which was higher than the zinc contents in other 120 food samples, as previously reported [11]. Based on dry weight, the mantle of the Pacific oyster *C. gigas* was reported to have the highest zinc content (2.01 ± 0.46 g/kg), followed by the gill (1.97 ± 0.28 g/kg), and the adductor muscle had the least (0.25 ± 0.04 g/kg) [13]. Zn content in the mantle was significantly higher than that in the gonadal-visceral mass and adductor muscle (*p* < 0.05) [22]. The result of this study demonstrates that oysters provide an excellent source of supplemental zinc. And the mantle and gill had a greater capacity for zinc accumulation than other tissues.

After using two kinds of gel filtration chromatograms, a protein with a molecular weight of approximately 36 kDa, was obtained. The prevailing belief is that intracellular zinc attaches to small proteins abundant in cysteine, commonly referred to as MT (<7000 Da), which effectively capture zinc in the presence of elevated concentrations. Nevertheless, in particular situations, it has the potential to be liberated for utilization by alternative proteins [23,24]. Oysters also yielded G-actin, a solitary polypeptide chain comprising 375 amino acids and a molecular weight of 42 kDa [25]. The zinc-binding protein MW used in our study is around 36 kDa. In contrast to the preceding study, which postulated it to be an innovative zinc-binding protein.

The 36-kDa protein was identified using a Q-Exactive mass spectrometer from *M. hongkongensis* and was deemed as MhCA. CA is a kind of zinc-containing metalloenzyme that exists widely in nature. In the past few decades, CAs have been verified to exist in a variety of invertebrate animals, including annelids, mollusks, arthropods, and cnidaria. CA activity was detected in the mantle, gill, muscle, and blood of mollusk, and some CAs have been shown to participate in the mobilization of calcium reserves in the mantle and ion transportation in the gill [26]. In the crystal structure of humans, there is a tetrahedral Zn^2+^ fissure at the bottom and an imidazole side chain of three histidine ligands located at positions 94, 96, and 119, which are known to be directly linked to the metal center [27]. To date, CAs from oysters *C. gigas* [20], *C. virginica*, and *O. edulis* (https://www.ncbi.nlm.nih.gov/genbank/, accessed on 5 February 2023) have been reported.

The presence of a signal peptide with 17 amino acid residues (^1^MMLSWLCLSSAILFTRA^17^) at the N-terminal of MhCA suggests its potential secretion to the periplasm, extracellular medium, or combination with the cell membrane [28]. The zinc ion-binding sites, which include three histidine residues (His^111^, His^113^, and His^136^), are recognized for their direct binding to the zinc ion [29]. The three histidine amino acids have been shown to be highly conserved in many studies. As an example, a CA gene was identified in *Penaeus monodon* by differential display PCR, and then the full-length cDNA sequence was isolated by RACE-PCR. The sequence analysis revealed that the three histidine amino acids were positioned at His^91^, His^93^, and His^116^, which make up the zinc-binding site [30]. Two CAs of *Litopenaeus vannamei* were isolated by RACE-PCR. The zinc-binding site consists of three histidine amino acid residues positioned at His^91^, His^93^, and His^116^ in LvCAc and His^115^, His^117^, and His^140^ in LvCAg [31]. The zinc-binding site consists of three histidine amino acid residues positioned at His^130^, His^132^, and His^155^ in HcCA3 from *H. cumingii* [32]. Notwithstanding the disparities in their locations of histidine amino acid residues, the distance between them remains identical, presumably owing to the necessity of folding space. Zinc ion-binding site residues are completely conserved, which may be the key to CA function. The homology between *M. hongkongensis* and *C. gigas* is the highest and a strict evolutionary relationship between the mollusk and the chordate can be found in the phylogenetic tree, which is consistent with other reported CA trees [20,33]. CA was evolutionarily conserved.

Analyzing the docking outcomes of two structural types using Swiss-model and AlphaFold2 with zinc ions, it is evident that these zinc ions mainly establish coordination bonds with six residues (His^111^, His^113^, His^136^, Ser^122^, His^124^, and His^129^) in the MhCA protein. The human CA II structure (4PXX) contained two bivalent metal ions and six residues (His^3^, Ser^5^, His^64^, His^94^, His^96^, and His^119^) in Swiss-model. Among them, the amino acid components were serine and histidine residues, with a greater ratio of histidine to serine residue. Histidine had a significant impact on zinc binding to the protein. Of all known protein structures, cysteine, glutamic acid, aspartic acid, and histidine make up around 96% of all residues that bind to zinc [34,35,36,37]. A MhCA protein could bind two zinc ions. The major amino acid residue was histidine residue.

## 4. Materials and Methods

### 4.1. Oyster and Tissue Sampling

Live oysters *M. hongkongensis* (shell length: 12 ± 1 cm, shell width: 9 ± 1 cm) were purchased from an oyster farm in Zhanjiang, China, in December 2022. The samples were transported to the laboratory in polystyrene boxes packed with crushed ice. Three oysters were selected for the cDNA cloning experiment and their soft tissues were removed and homogenized. Nine oysters were selected at random for the determination of zinc content, three individuals formed a replicate, and the experiment contained three replicates. The remaining oysters were subjected to protein extraction. Mantle, gill, visceral mass, and adductor tissues dissected from each individual were polled, immediately frozen in liquid nitrogen, and stored at −80 °C until needed.

### 4.2. Determination of Zinc Content from Different Tissues

ICP-MS analysis was utilized to ascertain the zinc levels in various tissues such as the mantle, visceral mass, gill, and adductor. The tissue samples were thoroughly dehydrated at 80 °C until they attained a consistent weight, which was documented. Approximately 100 mg of dried samples were digested thoroughly in a microwave digestion system with 5 mL concentrated HNO_3_ and 1 mL H_2_O_2_ under the following conditions: 25–120 °C, 5 min; 120–150 °C, 5 min; 150–185 °C, 20 min; and 185–70 °C, 20 min. Digested samples were diluted to a volume of 50 mL with 5% HNO_3_. Following that, the treated samples were subjected to ICP-MS analysis utilizing an Agilent 7500 (Agilent, Wilmington, DE, USA) apparatus. The display of zinc content in each tissue was carried out by μg/g, and the calculation of the proportion in each tissue was based on their dry weight.

### 4.3. Protein Extraction and Purification

#### 4.3.1. Extraction of Water-Soluble Proteins

The method for extracting WSPs was based on the established protocol, with slight modifications [38]. The tissues of the mantle, visceral mass, gill, and adductor were homogenized at 0 °C for 5 min (IKA, ULTRA-TUEEAX T-25) with 8 volumes of 50 mM phosphate buffer saline (PBS, pH 7.2). The homogenate was extracted at 12 °C for 90 min with constant stirring. The supernatant was collected after centrifugation at 22,000× *g* (4 °C, 20 min) (TDL-5-A, Shanghai, China). Finally, the supernatant extracted from different parts was filtered using a 0.45 µm syringe filter (ANPEL, Shanghai, China). Combined with the results of zinc content in each tissue, the crude WSPs from one of the oyster tissues with high zinc content were selected for the purification of a novel zinc-binding protein.

#### 4.3.2. Purification of Zinc-Binding Protein

Two types of gel filtration chromatograms were employed to purify a unique zinc-binding protein. The zinc concentration in protein fractions was monitored as a measure of zinc-binding protein purification. Firstly, the crude WSPs were separated into SuperdexTM 200 gel columns (2.6 × 65 cm) and eluted with 50 mM PBS at a flow rate of 2.0 mL/min, and the absorbance of the eluted fractions at 280 nm was monitored continuously with an ultraviolet spectrometer. The fractions of the same group were mixed and freeze-dried in a vacuum. The protein concentration was determined by the BCA kit. The zinc content of the protein fractions pooled was calculated by ICP-MS analysis and the MWs were verified by SDS-PAGE. Subsequently, the fractions containing the greatest amount of zinc were subjected to additional purification using a TSKgel G2000SWXL column (7.8 mm I.D × 30 cm, 5 μm, GE, Chicago, IL, USA) and eluted with ultrapure water at a flow rate of 0.7 mL/min. The absorbances of the eluted fractions at 280 nm were determined using an ultraviolet spectrometer, the protein concentration was determined using a BCA kit, the zinc concentration of the eluted fractions was determined using ICP-MS analysis, and the MWs of the eluted fractions were determined using SDS-PAGE. The fractions containing the greatest amount of zinc were gathered, concentrated, and kept at 4 °C for safekeeping.

### 4.4. Peptides Identification of Zinc-Binding Protein

The fraction exhibiting the greatest zinc concentration was subjected to trypsin digestion utilizing the technique described earlier with slight modification [39]. The fraction was lyophilized and enzymatic hydrolyzed in 40 µL trypsin solution at 37 °C for 16 to 18 h. Peptide mixtures after enzymatic hydrolyzed were injected onto a Zorbax 300SB-C18 peptide trap (Agilent Technologies, Wilmington, DE, USA), desalted in an auto-sampler, and then separated via reverse phase capillary high-performance liquid chromatography (HPLC), using an RP-C18 column (0.15 × 150 mm, Column Technology Inc., Fremont, CA, USA) equilibrated with 95% solution A (0.1% formic acid in water). The mobile phase consisted of different proportions of solution A and solution B (84% acetonitrile with 0.1% formic acid in water). The mixtures of trapped peptides were eluted in solution B with a gradient of 4–50%, 50–100%, and 100% for 15, 10, and 10 min, respectively. The sample was separated and desalinated via HPLC before being analyzed by tandem mass spectrometry on a Q-Exactive mass spectrometer (Thermo Fisher, Waltham, MA, USA) with an electroscope interface and operated in the positive ion mode. For the mass spectrometer, a full MS scan was used, followed by 10 MS/MS scans on the 10 most intense ions in the MS spectrum, with the following parameters: repeat count of 2; repeat length of 30 s; and exclusion duration of 90 s. The spray voltage was 2.5 kV and the capillary temperature was set to 400 °C. Raw MS/MS spectrum was applied to the MASCOT search (http://www.matrixscience.com, accessed on 1 January 2023, Matrix Science, London, UK) against the sequences of the zinc-binding protein that had been recorded in the database.

### 4.5. cDNA Cloning

Nucleotide sequence information of identified zinc-binding protein was obtained based on the previously established protocol with modifications [40]. The full-length cDNA was cloned using homology cloning and the RACE PCR approach.

#### 4.5.1. RNA Isolation and cDNA Synthesis

TRIzol reagent (Invitrogen, Waltham, MA, USA) was employed to isolate total RNA from the tissues. The A260/280 ratio and 1.0% glucose gel electrophoresis were utilized to evaluate the RNA’s quality, purity, and integrity. The StarScript II RT Mix with gDNA Remover (GenStar, Shenzhen, China) was utilized to synthesize the template in order to acquire the cDNA of a partial fragment sequence and validate it. The 5′ RACE kit (Sangon Biotech, Shanghai, China) was utilized to synthesize the template in order to acquire the cDNA of the 5′ RACE sequence. The SMARTer^TM^ RACE cDNA Amplification Kit (Clontech, Palo Alto, CA, USA) was utilized to create the template in order to acquire the cDNA of the 3′ RACE sequence.

#### 4.5.2. Full-Length cDNA Amplification and Cloning 

**Figure 8:**
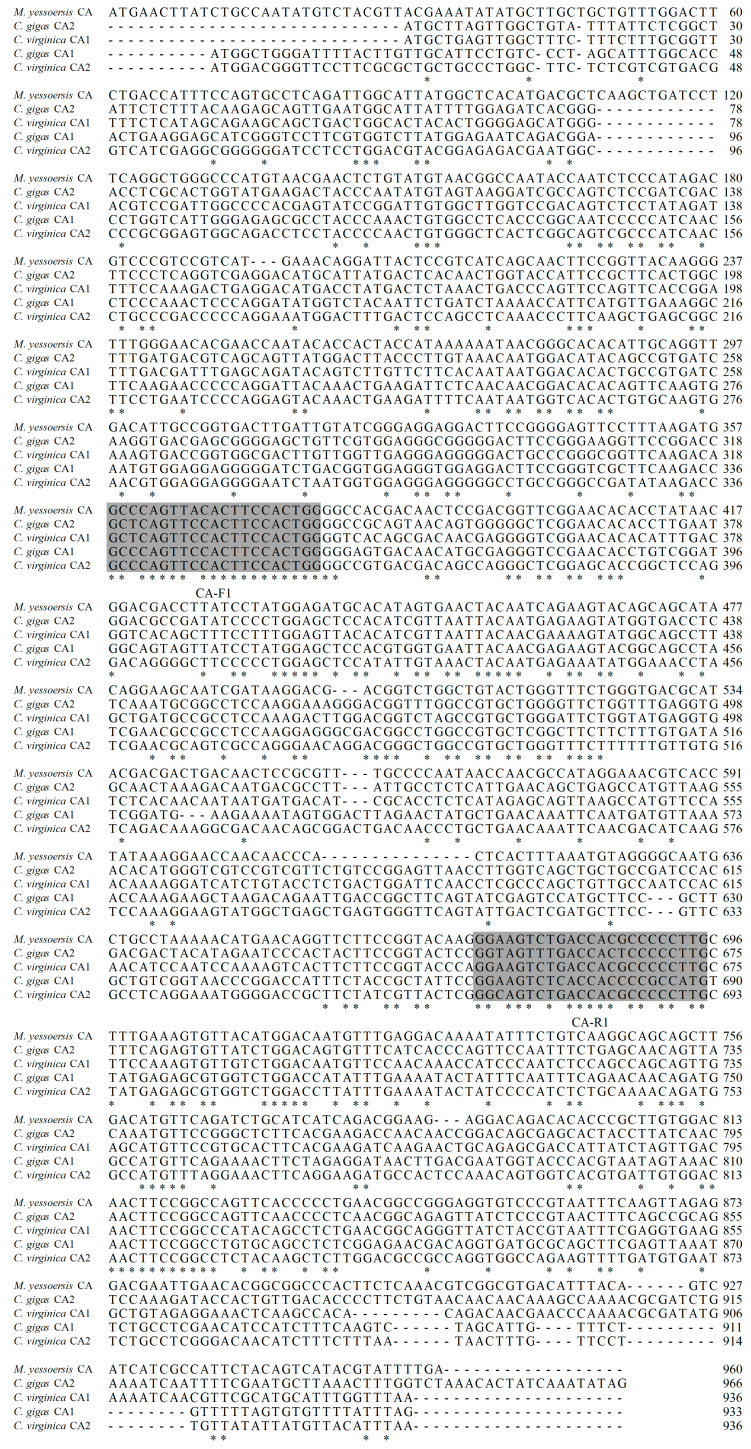
Schematic diagram of the design of the highly conserved region of the degenerate primers. Amino acid sequences alignments of *M. yessoensis* CA (XM_021517444), *C. gigas* CA1 (XM_011456734), *C. gigas* CA2 (XM_011436636), *C. virginica* CA1 (XM_022456657), and *C. virginica* CA2 (XM_022481449). The consistent amino acid residues in these five sequences are represented by *. Highly conserved regions are shaded in gray. The degenerate primers CA-F1/CA-R1 were designed based on highly conserved regions.

The same genes from different species have some conserved regions, which encode conserved residues. Therefore, two degenerate primers CA-F1 (5′-GCYCAGTTMCACTTCCACTGG-3′) and CA-R1 (5′-CAWGGSGGVGTGGTSARACT-3′) were designed after multiple alignments of the CA nucleotide sequence information among *M. yessoensis* CA (XM_021517444), *C. gigas* CA1 (XM_011456734), *C. gigas* CA2 (XM_011436636), *C. virginica* CA1 (XM_022456657), and *C. virginica* CA2 (XM_022481449) (Figure 8). Two degenerate primers CA-F1 and CA-R1 (Table 2) were used to obtain partial fragment sequence encodes the target protein. 

The polymerase chain reaction (PCR) was carried out using a 25 µL reaction volume containing 12.5 µL Go Taq^®^ Green Master Mix (Promega, Madison, WI, USA), 2 µL cDNA, 0.4 µM each primer, and ddH_2_O. PCR amplification was performed under the following conditions: 95 °C for 3 min, 30 cycles of 95 °C for 30 s, 55 °C for 30 s, 72 °C for 30 s, and 72 °C for 4 min. The PCR product was analyzed by 1.0% glucose gel electrophoresis. The target band was gel purified by Freeze ‘N Squeeze TM DNA Gel Extraction Spin Columns (Bio-Rad, Hercules, CA, USA), linked into the Pgem-T easy vector (Promega, Madison, WI, USA), and transfected into *E. coli* DH5α receptor cells and then inoculated onto LB solid medium (including AMP, X-gal, and IPTG). The positive colonies were detected by colony PCR using primers SP6 and T7 (Table 2) and then sequenced by Bioengineering (Shanghai) Co., Ltd. (Shanghai, China).

Based on the partial fragment sequence acquired using primers CA-F1 and CA-R1, four gene-specific primers CA-F2, CA-F3, CA-R2, and CA-R3 (Table 2) were designed using Primer Premier 5.0 software. To acquire the cDNA of the 5′ RACE sequence, primers 5-F-1 and CA-R2 were employed in the initial run. Subsequently, the appropriate amount of primary RACE products was utilized as the template for the second run, and amplified with primers 5-F-2 and CA-R3. To acquire the cDNA of the 3′ RACE sequence, primers 3-R-1 and CA-F2 were utilized in the initial run. Primers 3-R-2 and CA-F3 were employed to amplify the appropriate amount of primary RACE products as the template for the second run. Subsequently, the PCR products were gel purified and then sequenced as described above. 

Finally, the full-length cDNA sequence of the target protein was eventually obtained by overlapping the sequence data from the partial fragment, 5′ RACE and 3′ RACE. It was confirmed using primers CA-F4 and CA-R4 (Table 2) and then sequenced as described above.

### 4.6. Sequence Analysis

Analysis of the ORF was carried out using ORF Finder at NCBI (https://www.ncbi.nlm.nih.gov/orffinder/, accessed on 1 February 2023). The conserved domains present in the protein sequence were identified using Conserved Domains Search (https://www.ncbi.nlm.nih.gov/Structure/cdd/wrpsb.cgi, accessed on 1 February 2023). The sequence similarity was reported using the BLAST program (http://blast.ncbi.nlm.nih.gov/, accessed on 1 February 2023). The multiple alignment among CAs from *M. hongkongensis* and other species was performed using the ClustalW program (http://clustalw.ddbj.nig.ac.jp, accessed on 1 February 2023). Predictions of the isoelectric point and molecular weight of the amino acid sequence were performed using the ExPASY 3.0 (https://web.expasy.org/protparam/, accessed on 1 February 2023). Prediction of the signal peptide was carried out by the program SingalP 5.0 (https://services.healthtech.dtu.dk/service.php?SignalP-5.0, accessed on 1 February 2023). The phylogenetic NJ tree was constructed with the MEGA 7.0 software. To derive the confidence values for the phylogeny, bootstrap trials were replicated 1000 times [41].

### 4.7. Molecular Docking

Molecular docking analysis was performed using AutoDock Tools 4.2.6 through the following four steps. The zinc ion structure was first drawn using the Chewdraw program. Next, two structural experiments were performed using the SWISS-MODEL (http://swissmodel.expasy.org, accessed on 5 February 2023) and Colab AlphaFold2 (https://colab.research.google.com/github/sokrypton/ColabFold/blob/main/AlphaFold2.ipynb, accessed on 5 February 2024) to corroborate the 3D structure predictions of the protein MhCA. The quality of the models generated by Swiss-model was assessed by checking the stereochemical parameters using Procheck and Verify3D. In order to quantitatively measure the similarity between two protein structures, the RMSD value was calculated with two structures by overlapping them in PyMOL software. Third, the protein was utilized as a receptor, and the zinc ion was utilized as a ligand for molecular docking by identifying the grid box dimensions through the protein molecule as 40 × 40 × 40 with a spacing of 1 Å utilizing AutoDock Tools 4.2.6 software. The parameters of the protein were the default parameters, and the parameters of Zn^2+^ were field extensions of special force for Zn^2+^ in AutoDock Tools 4.2.6 software [42]. In total, 50 conformational structures were evaluated and ranked by energy, and the structures with the lowest evaluated score would be the best docking conformers. Finally, the results of molecular docking were analyzed by PyMOL 2.2.0 software (http://www.pymol.org, accessed on 7 February 2024).

### 4.8. Statistical Analyses

All data were expressed as the mean ± standard deviation (SD) of experiments carried out in triplicate (*n* = 3). Statistical analysis of the data was performed by one-way analysis of variance (ANOVA), followed by a test for significant difference (LSD) using SPSS 20.0. Differences were considered significant at *p* < 0.05. All experiments were performed in triplicate.

## 5. Conclusions

In this study, a 36-kDa zinc-binding protein with high zinc content from the mantle of *M. hongkongensis* was isolated, purified, and identified, which has been shown to be protein carbonic anhydrase. The full-length sequence of cDNA encodes the protein carbonic anhydrase, which is 1187 bp, and encodes 315 amino acids. Molecular docking results showed that two zinc ions could bind to the protein MhCA with six zinc-binding sites (His^111^, His^113^, Ser^122^, His^124^, His^129^, and His^136^).

## Figures and Tables

**Table 2:** Primers used in the present study.

Primer	Sequence (5′–3′)	Application
CA-F1 (forward)	GCYCAGTTMCACTTCCACTGG	Partial sequence
CA-R1 (reverse)	CAWGGSGGVGTGGTSARACT	
CA-R2 (reverse)	GCTCCAGTGGATATCGGT	5′ terminal sequence
CA-R3 (reverse)	GAGCCCTCGCTGTCACTG	
5-F-1 (forward)	GGCCACGCGTCGACTAGTACGGGIIGGGIIGGGIIG	
5-F-2 (forward)	GGCCACGCGTCGACTAGTAC	
CA-F2 (forward)	GAGGGCTCGGAACACACCTTGAATG	3′ terminal sequence
CA-F3 (forward)	GTATGGTGACCTCTCCAATGCGGC	
3-R-1 (reverse)	GCTGTCAACGATACGCTACGTAAC	
3-R-2 (reverse)	GCTACGTAACGGCATGACAGTG	
CA-F4 (forward)	CGGATTTTGTAGATCGGAGG	Complete sequence
CA-R4 (reverse)	GCATTTTATTGCTGTGATTT	
SP6 (forward)	ATTTAGGTGACACTATAG	Subclonal sequence
T7 (reverse)	GCTAGTTATTGCTCAGCGG	

B = G, T or C; M = A or C; N = A, C, G, or T; R = A or G; S = G or C; V = A, C, or G; Y = C or T, W = A or T.

## Data Availability

Data are contained within the article.

## Abbreviations

SDS-PAGE	sodium dodecyl sulfate–polyacrylamide gel electrophoresis
MhCA	carbonic anhydrase from *Magallana hongkongensis*
MT	metallothionein
WSP	water-soluble protein
MW	molecular weight
CA	carbonic anhydrase
PCR	polymerase chain reaction
ORF	open reading frame
RMSD	root mean square deviation
